# Mother–child conversations and coping strategies as antecedents of children’s recall accuracy

**DOI:** 10.3389/fpsyg.2025.1648952

**Published:** 2025-12-10

**Authors:** Seungjin Lee, Juyoung Kim

**Affiliations:** 1 Sanghuh College of Humanities, Konkuk University, Seoul, Republic of Korea; 2 Department of Psychological and Brain Sciences, The University of Iowa, Iowa, IA, United States

**Keywords:** free recall, suggestibility, mother–child conversations, coping strategies, stressful event

## Abstract

**Introduction:**

Considering that the level of stress during a distressing event can decrease children’s abilities to encode and store event-related information, and thereby their recall accuracy of the event, it is imperative to identify factors that can reduce the risk of high stress. This study explored cascading relations from mother–child conversations to children’s strategies for coping with stress to their recall accuracy regarding dental treatment to examine antecedents of recall accuracy related to a distressing event.

**Methods:**

Eighty 5- to 10-year-old children (41 boys, 39 girls) and their mothers participated in the study. Mother–child conversations and children’s coping strategies were, respectively, measured via coding based on mothers’ reports and children’s self-reports. Recall accuracy was assessed using the proportion of accurate free-recall reports and suggestibility in a memory interview.

**Results and discussion:**

Emotion-oriented conversations predicted higher recall accuracy (including more accurate free recall and less suggestibility) directly and indirectly through more use of positive coping strategies and less use of negative coping strategies. These results suggest that it is critical to consider the complex impacts of individual differences when studying children’s recall accuracy regarding stressful past events, especially in terms of interviewing child witnesses.

## Introduction

Children’s memory performance in stressful situations has been a focus of forensic research over the past few decades (see Deffenbacher et al., 2004; Marr et al., 2021 for a review). A question that continues to intrigue scholars is why some children recall stressful events accurately while others struggle with fragmented or distorted memories. One important aspect related to this variability is the intensity of stress felt during the event. High stress has been consistently reported to be related to poorer recall accuracy (e.g., Baker-Ward et al., 2015; Deffenbacher et al., 2004; Merritt et al., 1994; Wolf, 2017), although mild acute stress may temporarily facilitate memory performance (Deffenbacher et al., 2004). High levels of stress interfere with attention allocation and memory encoding, leading to poorer recall performance. Stress-induced impairments in memory encoding are especially problematic in contexts such as forensic interviews and medical assessments, where children’s recall and reports are crucial. Identifying factors that alleviate stress-related risks is therefore essential.

Children employ various strategies to manage stress and regulate negative responses (e.g., tension, anxiety, and fear) in distressing situations (Compas et al., 2001; Rodríguez et al., 2016; Schäfer et al., 2020). These coping skills can influence one’s amount of time spent managing stress, behavioral responses to stressors, and overall memories of the event (Aldwin, 2009). Researchers have defined dimensions of coping in several ways (e.g., Curry and Russ, 1985; Endler and Parker, 1990; Ryan, 1989), but the most popular and established categorization may be approach-oriented and avoidance-oriented strategies (Roth and Cohen, 1986). Approach-oriented strategies refer to individual efforts to deal with a stressful situation by actively facing and addressing the stressor, which include information seeking (i.e., asking questions or clarifying details about the event to reduce uncertainty), mood elevation (i.e., intentionally redirecting attention from the stressful event and inducing positive emotions such as joy, happiness, or pleasure), and cognitive restructuring or self-talk (Boals et al., 2008; Bray et al., 2019; Lee, 2011; Orvell et al., 2021; Peterson and Toler, 2013). These techniques are considered adaptive because they can facilitate recall by helping children focus on the experience rather than negative feelings, be less influenced by other people’s reactions, and reinterpret stressful situations in a more manageable way, thereby preserving cognitive resources for precise encoding and recall. Avoidance-oriented strategies, on the other hand, refer to individual attempts to distance themselves from the stressor, such as ignoring unpleasant stimuli or denying a stressor. Such techniques may relieve stress temporarily but not be adaptive in the long term, particularly for recall. They can weaken memory accuracy by preventing children from processing major details needed for adequate encoding and storage of the event (Goodman and Quas, 2018; Newman and Hedberg, 1999).

Given that approach-oriented strategies enhance recall accuracy by lowering stress and facilitating effective encoding and storage of event details, it is important to guide children in using such techniques during stressful situations. In this study, we investigated whether emotion-oriented mother–child conversations can shape children’s coping strategies, and, by extension, children’s subsequent recall accuracy. In general, children’s conversations with their parents can enhance the accuracy of the memory report by enabling children to practice elaborating their experiences, thoughts, and emotions. For instance, children whose mothers encouraged them to use detailed descriptions when discussing their experiences (including expressions on a variety of topics) have exhibited more accurate recall than children whose mothers did not (Fivush, 2019; McDonnell et al., 2016; Principe and London, 2022). This positive association has been shown to remain significant even after controlling for children’s language skills (Farrant and Reese, 2000; Reese and Newcombe, 2007). Thus, children with accumulated elaborative conversations with their parents may report an experienced event more coherently and accurately.

A limited perspective on cognitive resource allocation, which assumes that children possess finite cognitive and attentional resources that must be distributed across concurrent emotional and cognitive demands can provide a more fundamental framework explaining such a link from mother-child conversation to recall accuracy (Baddeley, 2020; Kahneman, 1973). During emotion-focused conversations, parents can help children perceive, label, and validate their feelings and learn suitable coping strategies through coaching and modeling (Gentzler et al., 2005; Gottman et al., 1996; Salmon and Reese, 2015). Thus, emotionally supportive mother–child conversations before a stressful event may help children regulate their affective arousal using more adaptive coping strategies, thereby freeing cognitive resources for effective memory encoding. Conversely, when emotional distress remains unresolved, a greater proportion of cognitive resources may be devoted to managing negative affect rather than encoding event details, leading to reduced recall accuracy.

For example, Gentzler et al. (2005) demonstrated that parent–child communication styles were related to children’s adaptive coping strategies (e.g., more reliance on support-seeking and cognitive decision-making and less on avoidant or aggressive strategies). Specifically, parents’ constructive reactions to the child (e.g., problem- and feeling-centered reactions, expressive encouragement, acceptance) and children’s affective sharing and emotion openness to the parent were related to more adaptive coping strategies, whereas parents’ unsupportive and distressing reactions were related to less use of adaptive coping strategies. Similarly, high-quality mother–child conversations, including maternal sensitive guidance and child cooperation, were related to approach-oriented coping, which in turn was associated with lower levels of angry feelings (Overbeek et al., 2022). This suggests that emotion-oriented mother–child conversations can lower children’s stress and negative feelings by helping them utilize more adaptive coping strategies. However, these studies measured parents’ and children’s general conversational styles during parent–child communications, not specific to conversations regarding upcoming stressful events.

We expect such relations would be particularly stronger for negative events because parents tend to be more descriptive and clearer in their narratives and to make more explicit causal links, when discussing negative events compared to positive ones (Fivush and Wang, 2005; Salmon and Reese, 2015). A growing body of evidence has demonstrated the associations between mother–child conversations and children’s recall of a past negative, stressful event, but most studies have examined the role of mother–child conversations *after* the event, not *before* the event, and the findings were mixed (see Lawson et al., 2018, for a review). Some studies have found that post-event parent–child discussions can facilitate the reliability and accuracy of children’s recall (e.g., Goodman et al., 1994; Peterson et al., 2007), while others have shown that conversations with parents can decrease the accuracy of recall or distort children’s memory (e.g., Alexander et al., 2010; Principe et al., 2017). Mother–child conversations before the event, however, may serve preparatory and regulatory functions, shaping children’s coping strategies and encoding processes before stressful experiences occur. Thus, emotion-oriented conversations discussing possible feelings and effective coping strategies would promote recall accuracy by helping children prepare for upcoming negative, stressful events. If conversations focus on anticipated unpleasant feelings and strategies to deal with them, then children may be able to identify their emotions more easily and apply proper techniques when facing a distressing event. Children should therefore better regulate their stress levels and encode event-based details more accurately.

Another important gap in the literature is that most studies have included events that children experienced with their mothers (e.g., Reese et al., 2019, 2020). However, in real-world situations such as investigative interviews, children are usually required to report details about an event that they did not experience with their mothers. As one exception, Baker-Ward et al. (2015) evaluated the relations between mother–child conversations and children’s memory accuracy regarding pediatric dental treatment, which children experienced alone (i.e., without a parent present). Children who talked about treatment more with their mothers before visiting the dentist demonstrated more accurate free recall and greater resistance to the interviewer’s misinformation than children who had fewer conversations with their mothers. However, the amount of conversation was determined based on mothers’ subjective ratings, which may introduce bias.

In sum, we examined mediation in order to understand a comprehensive pathway from mother–child conversations to children’s recall accuracy through coping strategies. We considered a naturally stressful event – a dental visit. A visit to the dentist is an authentic, often tense experience that can elicit anxiety and fear. It thus represents a useful paradigm for appraising children’s recall of a stressful personal event. To maintain objectivity, we rated mother–child conversations instead of relying on maternal self-reports as in previous studies. We anticipated a cascading relation from mother–child conversations to stress coping strategies to recall accuracy. That is, emotion-oriented conversations were expected to increase the accuracy of free recall and decrease suggestibility, namely by prompting children to use more positive and fewer negative coping techniques. While prior studies have examined some of these links separately, to our knowledge, the current study is the first to test all components simultaneously in a cascading pathway, which can provide a more comprehensive understanding of antecedents of children’s recall accuracy of stressful experiences.

## Method

### Participants

The participants included eighty 5- to 10-year-old children (41 boys, 39 girls) who were scheduled for general treatment (e.g., tooth decay, tooth extraction, sealant) at a pediatric dentistry clinic in Seoul, South Korea. The initial sample included 85 children, but data from five were excluded from analyses because those children either did not complete the memory interview or wanted to withdraw from the study. All remaining participants had visited the dentist more than twice and had received treatment from the same medical staff in this study more than once on average.

A sample size of more than 71 was deemed adequate for mediation using bias-corrected bootstrap with a power level of 0.80 and medium effects for both *a* path from the predictor to the mediator and *b* path from the mediator to the outcome (Fritz and MacKinnon, 2007). Therefore, our final sample size of 80 seemed sufficient.

### Procedure

In the clinic’s waiting area, we explained the study’s purpose and process to mothers and children and asked if they would be willing to participate in the study. For interested parties, we obtained verbal consent from children and informed consent from mothers. Then, while each child received treatment, their mother filled out a survey about her conversations with her child before the visit. Following a 10-min break after treatment, the child answered questions regarding stress coping strategies, reported their stress level during treatment, and completed memory interviews, which lasted roughly 20 min. Dental treatments took approximately 15 min on average (*M* = 14.15 min, *SD* = 4.35, range = 5–25 min), and differences in duration were not related to any study variables. The Institutional Review Board at Seoul National University approved this research (1212/001–002).

### Measures

#### Mother–child conversations

Mothers were asked to describe any conversations they had had with their children about the upcoming dental treatment—that is, whether they had discussed the visit in advance and, if so, what specific topics or messages had been shared. The survey was completed while mothers were waiting in the reception area during their child’s dental appointment. Based on the mothers’ written descriptions, two trained researchers independently coded the responses to determine (a) whether the conversation involved references to negative emotions (e.g., anticipated pain, tension, fear, or stress) related to the upcoming procedure, and (b) whether it included methods to regulate or cope with those emotions. Children whose mothers reported discussing strategies to help manage negative emotions (e.g., reassurance, comfort, encouragement, or relaxation techniques) were classified into the emotion-oriented conversation group (coded as 1), whereas those whose mothers’ conversations focused solely on factual or procedural information (e.g., what the dentist would do, why treatment was necessary) or who reported having had no discussion about the visit were assigned to the non-emotion-oriented conversation group (coded as 0). Each group consisted of 40 children. Coding reliability between the two raters was high (*κ* = 0.92).

Emotion-oriented conversations typically included emotionally supportive content designed to reduce the child’s anxiety or distress (e.g., “It might be scary, but it will be over soon and the dentist will help you”), whereas non-emotion-oriented conversations emphasized concrete or educational information about the procedure (e.g., “The dentist will clean your teeth and check for cavities”). The coding framework and classification criteria were guided by the procedures described in Baker-Ward et al. (2015) and Lee (2011), which demonstrated the methodological validity of coding based on parental self-reports to assess mother–child conversational content in relation to coping and emotion regulation. See Supplementary Table S1 for detailed coding criteria and representative examples of emotion-oriented and non-emotion-oriented conversations.

#### Stress coping strategies

Children’s stress coping strategies were assessed with 15 questions based on the Kidcope measure (Spirito et al., 1988) and the How I Coped under Pressure Scale (Ayers et al., 1996). All items were scored on a 4-point Likert-type scale and were classified as either positive or negative strategies following Baker-Ward et al. (2009). Positive strategies for coping with stress during medical treatment included mood elevation (3 items; e.g., “I told myself that my visit to the dentist would be over soon.”), social support (3 items; e.g., “I held someone’s hand so I would feel better.”), and information seeking (2 items; e.g., “I asked lots of questions, so I would know just what the dentist was doing.”). Negative strategies included active escape (3 items; e.g., “I did something to try to get away like jumping out of the chair.”), avoidant behavior (3 items; e.g., “I wished I wasn’t at the dentist anymore.”), and resignation (1 item; e.g., “I did not do anything. Nothing would have helped.”). We asked children if they used these strategies to cope with pain or stress during treatment and, if so, how often (1 = *not at all*, 2 = *a little*, 3 = *a lot*, 4 = *always throughout treatment*). We then calculated the average of each coping strategy, with a high score indicating more use.

#### Recall accuracy

We evaluated children’s recall accuracy through a memory interview consisting of questions about the terms and tools frequently mentioned or used during treatment (for detailed procedures, see Baker-Ward et al., 2015; Lee, 2011). The questions were developed under pediatric dentists’ guidance. Each interview proceeded hierarchically, wherein questions were posed based on the child’s responses (i.e., starting with an open-ended question, followed by semi-open-ended questions, and finally yes/no questions; La Rooy et al., 2015). Hierarchical interviews are known to improve children’s accuracy when recalling experiences by encouraging voluntary reporting while minimizing misleading or suggestive information provided by an interviewer (Benia et al., 2015).

Each interview started with an open-ended question, “Would you like to tell me all the things you remember from the dental treatment today?” If the child provided short responses without sufficient details (e.g., “The dentist made me hurt,” “The dentist treated me”), the interviewer asked semi-open-ended questions in the form of ‘how’ or ‘what’ (e.g., “How did the dentist hurt you?,” “What did the dentist treat you with?”) to prompt more specific answers. Lastly, yes/no questions covered what the child had and had not experienced during treatment. Questions about unexperienced events were meant to test the child’s susceptibility or resistance to suggestive questions (e.g., “Did the dentist give you candy after treatment as a reward?”).

We quantified the accuracy of children’s responses to the interviewer’s questions by counting the number of correct answers, following a system described in Lamb et al. (1996). The accuracy of children’s responses was evaluated against the treatment records and dentist reports to ensure objective identification of correct answers. Items that were mentioned repeatedly were not counted more than once. Among the questions a child answered correctly, those that were in response to open-ended and semi-open-ended questions were coded as *free recall*. We calculated a free-recall score by dividing the number of correctly answered open-ended and semi-open-ended questions by the total number of those questions, with higher scores indicating more accurate free recall. In addition, if the child incorrectly responded “yes” to a yes/no question about something that did not occur during treatment, this answer was coded as *suggestibility*. A suggestibility score was calculated by dividing the number of incorrect “yes” responses by the total number of suggestive questions asked; a higher suggestibility score demonstrated that more suggestive questions were incorrectly answered with “yes.” Overall, the higher the child’s free-recall scores and the lower their suggestibility scores, the better the child’s recall accuracy. Interrater reliability, measured with the kappa statistic, was 0.90 for free recall and 0.95 for suggestibility.

## Results

### Preliminary analyses

To ensure that the use of positive and negative coping strategies was indeed, respectively, related to the decrease and increase in the level of stress during treatment, we checked correlations between coping strategies and the level of stress, measured by physiological stress responses, observer-rated stress, and child self-report (see Supplementary Material 1 for the details of stress measures). More use of positive coping strategies was related to lower stress measured by observer rate (*r* = −0.46, *p* < 0.001) and child self-report (*r* = −0.29, *p* < 0.01), but not to physiological stress response (*r* = −0.17, *p =* 0.13). More use of negative coping strategies was related to higher stress measured by all three measures (*r* = 0.23, *p* < 0.05 for physiological response, *r* = 0.54, *p* < 0.001 for observer rate, *r* = 0.46, *p* < 0.001 for child self-report). Also, associations between higher stress and lower recall accuracy were supported (*r* = −0.77 – −0.23, *p* < 0.05, 0.01 or 0.001 for free recall, *r* = 0.32–0.69, *p* < 0.01 or 0.001 for suggestibility).

### Correlations

Descriptive statistics and correlations were computed in SPSS 26.0 (IBM Corp., 2019; Tables 1, 2). Mother–child conversational styles and children’s coping strategies were significantly related to free recall and suggestibility. Children who had emotion-oriented conversations with their mothers before treatment were likely to exhibit more accurate free recall and lower suggestibility compared with children who had non-emotion-oriented conversations. Children who more actively employed positive coping strategies during treatment displayed more accurate free recall and lower suggestibility. The relations of negative coping strategies with free recall and suggestibility showed opposite patterns: heavier use of negative coping strategies was related to less accurate free recall and higher suggestibility. Mother–child conversational styles and children’s coping strategies also correlated with each other. Emotion-oriented conversations were related to more use of positive coping strategies and less use of negative coping strategies. However, positive and negative strategies did not have significant correlations with each other. Child gender was not significantly related to any of the variables.

**Table 1:** Descriptive statistics of study variables.

Variables	Mean	Standard deviation	Range
Child age in months	93.74	18.71	61–130
Positive coping strategies	2.00	0.57	1.00–3.50
Mood elevation	1.75	0.81	1.00–4.00
Social support	2.11	0.82	1.00–4.00
Information seeking	2.12	0.79	1.00–3.50
Negative coping strategies	1.35	0.48	1.00–3.78
Active escape	1.23	0.67	1.00–4.00
Avoidant behavior	1.60	0.63	1.00–3.67
Resignation	1.25	0.80	1.00–4.00
Recall accuracy			
Free recall	0.34	0.18	0.07–0.88
Suggestibility	0.11	0.12	0.00–0.79

**Table 2:** Correlations among study variables.

Variables	Mother–child conversations	Positive coping strategies	Negative coping strategies	Free recall
Positive coping strategies	0.61^***^			
Negative coping strategies	−0.23^*^	−0.15		
Free recall	0.43^***^	0.55^***^	−0.38^***^	
Suggestibility	−0.34^**^	−0.42^***^	0.33^**^	−0.78^***^

Mother–child conversations were coded as 0 = non-emotion-oriented and 1 = emotion-oriented.

^*^p < 0.05, ^**^p < 0.01, ^***^p < 0.001.

### Relations from mother–child conversations to coping strategies to stress to recall accuracy

Indirect relations were tested in Mplus 7 (Muthén and Muthén, 1998-2012) with bootstrapping (5,000 samples) and bias-corrected 95% confidence intervals (CIs). Suggestibility scores were log-transformed due to high kurtosis. Given the limited sample size and non-significant correlations between positive and negative coping strategies, we created two separate models with each coping strategy as the mediator. Thus, we included dummy-coded emotion-oriented mother–child conversations as the predictor, positive or negative coping strategies as the mediator, free recall accuracy and suggestibility as the outcomes, and children’s age as a covariate.

**Figure 1:**
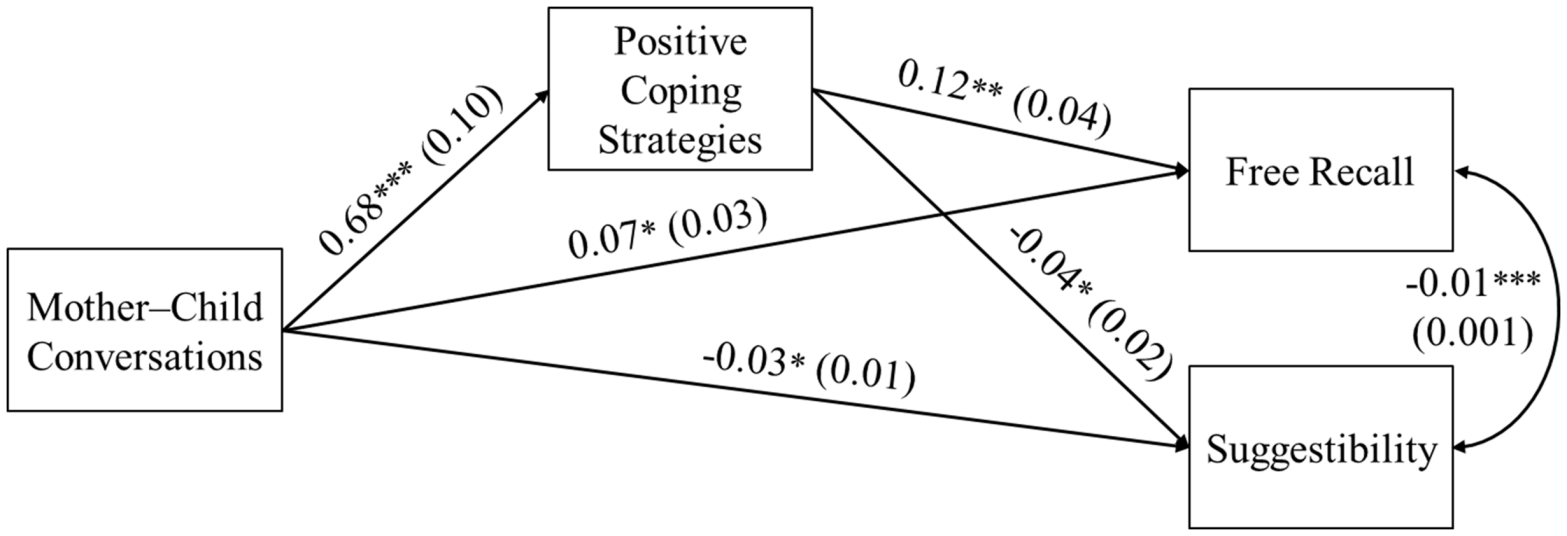
Relations from mother–child conversations to children’s recall accuracy via positive coping strategies. Unstandardized coefficients and standard errors in parentheses are presented. Mother–child conversations were coded as 0 = non-emotion-oriented and 1 = emotion-oriented. Child age was covaried and significantly related to free recall and suggestibility, but it is not shown in this figure for clarity. ^*^p < 0.05, ^**^p < 0.01, ^***^p < 0.001.

Figure 1 displays the results of the model with positive coping strategies as the mediator. Emotion-oriented mother–child conversations predicted more use of positive coping strategies, which in turn was related to better free recall (*B* = 0.08, *SE* = 0.04, 95% CI: [0.011, 0.155]) and lower suggestibility (*B* = −0.03, *SE* = 0.02, 95% CI: [−0.062, −0.005]). The direct paths from mother–child conversations to free recall and suggestibility were significant.

**Figure 2:**
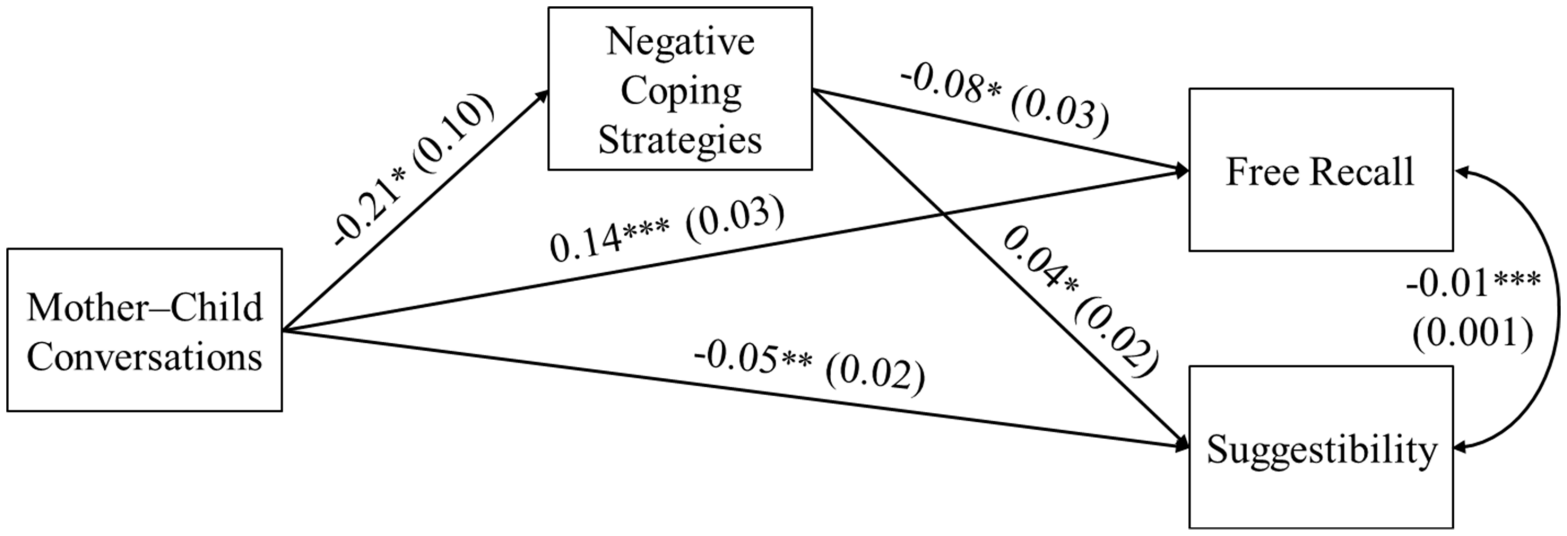
Relations from mother–child conversations to children’s recall accuracy via negative coping strategies. Unstandardized coefficients and standard errors in parentheses are presented. Mother–child conversations were coded as 0 = non-emotion-oriented and 1 = emotion-oriented. Child age was covaried and significantly related to free recall and suggestibility, but it is not shown in this figure for clarity. ^*^p < 0.05, ^**^p < 0.01, ^***^p < 0.001.

We found similar patterns of results for the model with negative coping strategies as the mediator (Figure 2). Emotion-oriented mother–child conversations predicted higher free-recall accuracy and lower suggestibility through less use of negative coping strategies (*B* = 0.02, *SE* = 0.01, 95% CI: [0.003, 0.035] for free recall; *B* = −0.01, *SE* = 0.004, 95% CI: [−0.018, −0.001] for suggestibility). The direct paths from mother–child conversations to free recall and suggestibility were again significant.

### Sensitivity analyses

Given that our sample size justification was based on a mediation model with one outcome, we estimated models for each outcome, instead of having two outcomes in the same model. The coefficient values and mediation results in the separate models were almost identical to those in the models with the two outcomes.

## Discussion

In the current study, we sought to identify aspects related to children’s abilities to accurately recall and report a past stressful event. In child abuse cases, the child and the alleged perpetrator are typically the only witnesses to the event, and the child is often required to elaborate on witnessed or experienced acts of violence or accidents. Thus, given that the child’s capacity to serve as a reliable witness is of paramount importance, it is essential to understand the factors associated with their abilities to recall and report stressful personal experiences.

We investigated mother–child conversational styles and children’s stress coping strategies as antecedents of recall accuracy in 5- to 10-year-old South Korean children. Our findings supported significant associations between emotion-oriented mother–child conversations and higher recall accuracy via more proper use of coping strategies. In particular, children who had emotion-oriented conversations with their mothers before treatment gave more accurate reports about treatment and were less susceptible to misinformation through more use of positive coping strategies and less use of negative coping strategies. Therefore, alleviating stress by employing proper coping strategies is imperative for accurate recall and reports as expected. Parental efforts to help children use such strategies (e.g., having conversations about anticipated feelings and emotions beforehand, reminding children to refrain from using negative coping strategies) can indeed prompt more precise free recall and stronger resistance to interviewers’ suggestive questions.

Although some scholars have argued that emotion-oriented conversations held *after* an event could distort children’s memories and diminish the accuracy of event-related recall (Lawson et al., 2018; Principe and London, 2022), we found that having emotion-oriented conversations *before* a stressful event could promote accurate recall and reports. If a child is prepared in advance (i.e., through relevant conversations with their mother) for negative emotions they may experience later, the child can better regulate negative emotions and apply more effective and positive strategies or refrain from less adaptive strategies to cope with stress during an event (Wu et al., 2020; Zinsser et al., 2021). Children equipped with positive coping strategies can have higher memory accuracy because they can allocate cognitive resources more efficiently. That is, their cognitive resources are sufficient for processing information because these children do not need to devote an excessive volume of resources to controlling negative emotions (Alexander et al., 2002; Peterson et al., 2007). By contrast, children with negative coping strategies may find it difficult to store event-related information: being overwhelmed by negative emotions can exacerbate their stress levels during the event and prevent them from using their cognitive resources to process incoming information appropriately.

Based on this study’s findings, creating developmentally and individually sensitive guidelines for interviewing children in the legal system is recommended. Investigative interviewers should consider ways to help children utilize adaptive, approach-oriented coping strategies (e.g., mood elevation, information seeking, social support) while discouraging the use of negative, avoidant-oriented coping strategies (e.g., distraction, escape, and denial) to reduce anxiety and stress before conducting an interview. Given that emotion-oriented conversations between mothers and children were effective in the current research, freely talking about children’s emotions and sharing examples of coping strategies during a rapport-building phase before a substantive phase of an interview is considered vital. It could enhance children’s recall accuracy and prepare them to resist potentially misleading information from interviewers. Moreover, guiding children to use approach-oriented coping techniques and have conversations beforehand may be applicable in other stressful environments, such as clinical settings, especially for children who are intensely afraid of treatment due to past medical experiences that evoked severe negative feelings. Advocating for these strategies could help not only children but also medical staff to proceed smoothly with treatment.

This study is not without limitations. Although we tried to measure the constructs in a valid and reliable manner, children’s coping strategies were self-reported. This approach seemed most suitable because certain measures of implicit strategies were only available from the child (e.g., “I told myself that my visit to the dentist would be over soon.”). However, future research could integrate methods such as self-reports and observations to increase measurement validity. For mother–child conversations, we used systematic content-coding based on maternal reports, but future research with direct observation, which can provide more detailed information (e.g., frequency of emotional words, discussion of causes and outcomes of emotions, etc.), would strengthen methodological rigor and ecological validity. Also, we did not include the level of stress during treatment as part of a cascade, given our limited sample size and its well-established associations with coping strategies and recall accuracy. However, correlation results supported these associations. Future research with a larger sample, ideally in a longitudinal and experimental design, is needed to clarify the temporal and causal dynamics among mother–child conversations, coping strategies, stress, and recall accuracy.

Other unmeasured personal and contextual characteristics might further explain our identified mechanism or serve as a moderator. For example, although we focused on that mother–child conversations would help children use more adaptive coping strategies, the relations can be bidirectional, such that their general stress coping skills can influence their conversational styles (Fivush and Sales, 2006). Additionally, mechanisms could be different depending on child age or family socioeconomic status (SES). Since differences by age or SES were not the main focus of the study and our sample size was limited, we did not test whether these variables moderated any of the relations among mother–child conversations, stress coping strategies, and recall accuracy. Note, however, that we covaried child age and found that older children were less likely to use negative coping strategies and had better free recall and lower suggestibility. Older children have advanced cognitive skills due to linguistic and metacognitive maturity, which may enable them to employ better coping strategies, more effectively process information, and more accurately remember and report the details of the past event (e.g., Compas et al., 2001; Geddie et al., 2000). Also, children’s coping could be different by SES as the effectiveness of coping strategies could depend on context. Future research with a larger sample would be warranted to examine differences by age or SES in the effects of emotion-focused conversations and coping strategies on recall accuracy, to identify which developmental period is most sensitive to emotion-focused conversations and different coping strategies, and to explore if the patterns differ by family SES.

Additionally, cultural backgrounds can be an interesting moderator. For instance, U. S. mothers were more likely to attempt to understand children’s feelings and elaborate on the cause of those feelings, whereas Chinese mothers used more negative emotional words and employed a more directive and disciplinary approach by emphasizing proper behaviors (Fivush and Wang, 2005; Wang and Fivush, 2005). It not only highlights cultural differences in mother–child conversational styles and children’s stress coping strategies but also suggests that our findings from South Korean children may not be applicable to children with different cultural backgrounds.

In conclusion, we discovered several tactics that could facilitate the accuracy of children’s reports of a stressful past event (e.g., pre-event emotion-oriented mother-child conversations and less use of negative, avoidance-oriented coping strategies). The current findings can further strengthen ecological validity and generalizability to real-world forensic and medical settings because the study concerned a naturally distressing event (i.e., pediatric dental treatment). By identifying pre-event conversations and stress coping as critical antecedents of recall performance, this research integrates emotion socialization and cognitive stress-memory frameworks. Based on the findings, we would advise researchers studying children’s memories of stressful events to account for the complex effects of numerous characteristics instead of simply relying on children’s stress levels.

## Data Availability

The raw data supporting the conclusions of this article will be made available by the authors, without undue reservation.
