# Supplementary material for: Mother–child conversations and coping strategies as antecedents of children’s recall accuracy
Source: Front Psychol. 2025 Dec 10;16:1648952. doi: 10.3389/fpsyg.2025.1648952 (PMC12727546; doi:10.3389/fpsyg.2025.1648952)
Supplement: Supplementary file 1 [file Table_1.docx]

Mother-Child Conversations and Coping Strategies as Antecedents of Children's Recall Accuracy

**Table S1. Examples and Coding Details for Mother–Child Conversations**

| Category | Operational Definition | Typical Conversational Features | Example of Mother’s Written Response (Anonymized) | Coding Notes / Criteria |
| --- | --- | --- | --- | --- |
| Emotion-Oriented Conversation | Conversation includes explicit references to the child’s emotions (e.g., fear, anxiety, worry) and/or strategies for emotional regulation before the dental visit. | Emotional reassurance, comfort, encouragement, coping suggestions (e.g., deep breathing, normalization). | “My child seemed nervous before the appointment, so I told her, ‘It might be scary, but it will be over soon and the dentist will help you.’” “I said, ‘Don’t worry, Mommy will be right here, and you can hold my hand if you’re scared.’” | Must contain at least one reference to negative emotion and one coping/regulation strategy. If both elements are present, classify as emotion-oriented. |
| Non-Emotion-Oriented Conversation | Conversation focuses on factual, procedural, or educational content without emotional language or coping guidance. | Descriptions of the dental procedure, health-related information, behavioral instructions. | “I told my child that the dentist would clean and check for cavities.” “I explained that we were going to finish the filling we started last time.” | Includes no mention of emotion. Focused solely on factual or procedural content. If no conversation was reported, classify as non-emotion-oriented. |

This supplementary table provides detailed coding criteria, operational definitions, and representative examples of emotion-oriented and non-emotion-oriented mother–child conversations, as used in the current study. These details are provided to enhance methodological transparency and replicability.

**Stress Measures**

***Physiological Stress Response***

We measured children’s blood pressure before and after dental treatment using an electronic sphygmomanometer (Omron HEM-780). Mean blood pressure was calculated from each child’s diastolic and systolic blood pressure values, following the method of Zheng et al. (2008). The change in mean blood pressure before and after treatment was used as an indicator of the child’s stress level during treatment; the higher the change in mean blood pressure, the greater the shift (Boyce et al., 1995). The mean was 5.60 (*SD* = 9.35, Range = -20 – 40). A positive change in mean blood pressure represented an increase in stress during treatment, whereas a negative change conveyed a reduction in stress.

***Observer-Rated Stress***

Medical staff evaluated each child’s level of stress based on the child’s behavior during treatment using the Frankl scale from 1 to 4 (1 = *the child appears to have experienced extreme stress during treatment*, 4 = *the child appears to be very cooperative with treatment and has not experienced any stress due to treatment*). This scale is common when tracking children’s stress levels in the context of pediatric dentistry (Frankl et al., 1962). The mean was 3.13 (*SD* = 0.58, Range = 1 – 4). Because a lower score represents greater stress, we used a reverse-coded rating to consistently interpret higher scores as indicative of higher stress.

***Child’s Self-Reported Stress***

Children’s self-assessments of their stress during treatment were obtained via the Visual Analog Scale (VAS; Shields et al., 2003), which includes a picture in the shape of a thermometer (1 = *not afraid of the treatment at all* *or* *not hurt at all* to 5 = *very scared of the treatment* *or* *very hurt*). Children were asked to physically point to the level of stress they had experienced during treatment. The mean was 2.06 (*SD* = 1.29, Range = 1 – 5). The VAS is a popular tool for measuring children’s stress levels in medical settings that may evoke anxiety or fear (Lin et al., 2017).

**Coding Procedure Summary**

This supplementary document provides transparency regarding how maternal reports were interpreted and classified. It illustrates the coding logic, operational definitions, and representative examples of each category to support reproducibility and methodological clarity.

• Coding team: Two trained coders independently reviewed all maternal written responses (*N* = 80).
• Coding criteria: Each response was evaluated on two dimensions: (a) mention of negative emotions, and (b) mention of coping/regulation strategies.
• Decision rules:
 - If both (a) and (b) were present → Emotion-oriented conversation
 - If neither was present → Non-emotion-oriented conversation
• Interrater reliability: Cohen’s κ = .92 (excellent agreement).
• Discrepancies: Resolved through discussion until consensus was reached.

**References**

Boyce, W. T., Chesney, M., Abbey, A., Tschann, J., Adams, S., Chesterman, B., Cohen, F., Kaiser, P., Folkman, S., & Wara, D. (1995). Psychobiologic reactivity to stress and childhood respiratory illness: Results of two prospective studies. *Psychomatic Medicine, 57*(5), 411-422.

Frankl, S., Shiere, E, & Fogels, H. (1962). Should the parent remain with the child in the dental operatory? *Journal of Dentistry for Children, 29*, 150–163.

Lin, C. S., Wu, S. Y., & Yi, C. A. (2017). Association between anxiety and pain in dental treatment: A systematic review and meta-analysis. *Journal of Dental Research, 96*(2), 153–162. https://doi.org/10.1177/0022034516678168

Shields, B. J., Palermo, T. M., Powers, J. D., Grewe, S. D., & Smith, G. A. (2003). Predictors of a child's ability to use a visual analogue scale. *Child: Care, Health and Development*, *29*(4), 281-290. https://doi.org/10.1046/j.1365-2214.2003.00343.x

Zheng, L., Sun, Z., Li, J., Zhang, R., Zhang, X., Liu, S., Li, J., Xu, C., Hu, D., & Sun, Y. (2008). Pulse pressure and mean arterial pressure in relation to ischemic stroke among patients with uncontrolled hypertension in rural areas of China. *Stroke, 39*(7), 1932-1937. https://doi.org/10.1161/STROKEAHA.107.510677
